# Inhibition of lipid metabolism exerts antitumor effects on rhabdomyosarcoma

**DOI:** 10.1002/cam4.4185

**Published:** 2021-09-02

**Authors:** Satoshi Miyagaki, Ken Kikuchi, Jun Mori, Gary D. Lopaschuk, Tomoko Iehara, Hajime Hosoi

**Affiliations:** ^1^ Department of Pediatrics Graduate School of Medical Science Kyoto Prefectural University of Medicine Kyoto Japan; ^2^ Department of Pediatrics Uji Takeda Hospital Kyoto Japan; ^3^ Department of Pediatrics Mazankowski Alberta Heart Institute University of Alberta Edmonton Canada

**Keywords:** cancer metabolism, lipid metabolism inhibition, low‐fat diet, malonyl‐CoA decarboxylase inhibitor, rhabdomyosarcoma

## Abstract

Rhabdomyosarcoma exhibits tumor‐specific energy metabolic changes that include the Warburg effect. Since targeting cancer metabolism is a promising therapeutic approach, we examined the antitumor effects of suppressing lipid metabolism in rhabdomyosarcoma. We suppressed lipid metabolism in rhabdomyosarcoma cells in vitro by administering an inhibitor of malonyl‐CoA decarboxylase, which increases malonyl‐CoA and decreases fatty acid oxidation. Suppression of lipid metabolism in rhabdomyosarcoma cells decreased cell proliferation by inducing cell cycle arrest. Metabolomic analysis showed an increase in glycolysis and inactivation of the pentose phosphate pathway. Immunoblotting analysis revealed upregulated expression of the autophagy marker LC3A/B‐II due to increased phosphorylation of AMP‐activated protein kinase, a nutrient sensor. p21 protein expression level also increased. Inhibition of both lipid metabolism and autophagy suppressed tumor proliferation and increased apoptosis. In vivo studies involved injection of human Rh30 cells into the gastrocnemius muscle of 6‐week‐old female nude mice, which were divided into normal chow and low‐fat diet groups. The mice fed a low‐fat diet for 21 days showed reduced tumor growth compared to normal chow diet‐fed mice. Suppression of lipid metabolism disrupted the equilibrium of the cancer‐specific metabolism in rhabdomyosarcoma, resulting in a tumor growth‐inhibition effect. Therefore, the development of treatments focusing on the lipid dependence of rhabdomyosarcoma is highly promising.

## Graphical abstract

Rhabdomyosarcoma relies on lipid metabolism to maintain its growth. Inhibition of lipid metabolism exerts antitumor effects via disrupting the equilibrium of cancer‐specific energy metabolism. The development of treatments focusing on the lipid dependence of rhabdomyosarcoma is highly promising.

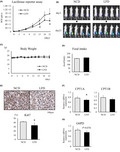

## 1 INTRODUCTION

Rhabdomyosarcoma (RMS), the most prevalent malignancy among pediatric soft tissue tumors,^1^, ^2^, ^3^ arises from myogenic mesenchymal progenitors and occurs in various organs and tissues such as the bladder, gonads, nasopharyngeal cavity, paranasal sinuses, parameninges, orbit, and skeletal muscle.^4^, ^5^ While recent progress in combined modality therapy has slightly improved the prognosis of patients with RMS, long‐term organ damage due to treatment‐related toxicity has become a serious concern. Therefore, there is an urgent need to develop tumor‐specific therapies with few side effects.

Rapidly proliferating cancer cells, especially solid tumor cells, are prone to hypoxia and nutrient starvation, resulting in metabolic reprogramming to maintain their enormous energy demands. It is widely known that glucose uptake is higher in cancer cells than in normal cells. In addition, cancer cells synthesize ATP primarily via glycolysis in the cytoplasm, which does not require oxygen, rather than oxidative phosphorylation in mitochondria, even in an oxygen‐rich environment. This phenomenon was first proposed by Otto Warburg approximately 100 years ago, and later named as the Warburg effect or “aerobic glycolysis.”^6^, ^7^ Moreover, cancer cells activate the pentose phosphate pathway (PPP), a branched pathway from glycolysis, to increase nucleic acid supply in order to maintain their rapid proliferative capacity, and NADPH synthesis to cope with oxidative stress.^8^, ^9^ It has been reported that glycolysis is significantly upregulated compared with normal myocytes in the alveolar‐type RMS cell line Rh30.^10^ Pyruvate kinase M (Pkm) is involved in the synthesis of pyruvate, the final product of glycolysis, and has two splicing variants, Pkm1 and Pkm2.^11^ Pkm1‐derived pyruvate is preferentially destined for the mitochondrial tricarboxylic acid (TCA) cycle, while Pkm2‐derived pyruvate is predominantly destined for lactate production. Most cancer cells significantly express Pkm2, and Rh30 is no exception.^12^ Therefore, the glucose taken up by RMS cells is mostly used for lactate synthesis, not glucose oxidation in the mitochondria. However, at the same time, it has been shown that the mitochondrial TCA cycle in Rh30 is enhanced, suggesting that energy substrates other than glucose, such as glutamic acid and fatty acids, may be oxidized.^10^ Recently, it has been reported that lipids are indispensable in some types of cancer cells, such as breast and prostate cancer cells.^13^, ^14^ Both lipid anabolism and catabolism are important metabolic pathways, with lipid anabolism (such as energy storage and lipid synthesis) being essential for cell membrane construction, while lipid catabolism (such as ATP synthesis by fatty acid oxidation) being critical for energy generation.

Thus, we hypothesized that suppression of lipid metabolism in RMS exerts an antitumor effect through an imbalance in cancer‐specific energy metabolism. In the present study, we verified this hypothesis using both in vitro and in vivo experiments.

## 2 MATERIALS AND METHODS

### 2.1 Cell culture

We used the following human alveolar RMS (ARMS) cell lines: SJ‐Rh30 (Rh30)^15^ and Rh41 (kindly provided by Peter J. Houghton, M.D.; Greehey Children's Cancer Research Institute, University of Texas Health Science Center, San Antonio TX, USA)^16^ and the human embryonal RMS (ERMS) cell lines, RD (Japanese Collection of Research Bioresources Cell Bank), and KP‐RMS‐KH (KH).^17^, ^18^, ^19^ Cells were cultured in DMEM supplemented with 1% FBS and 1% penicillin‐streptomycin at 37℃ in a 5% CO_2_ incubator; 1% FBS was used to imitate the living environment typical of various solid tumors, where nutrients and growth factors are likely to be deficient due to the inadequate vascularization.

### 2.2 Animals and diets

Five‐week‐old female BALB/c nu/nu nude mice (*n* = 10, bodyweight 16–18 g) were purchased from Japan SLC, Inc. (Hamamatsu, Japan). All experiments and procedures were conducted in accordance with the Institutional Animal Care and Use Committee guidelines. Animal experiments were approved by the Committee for Animal Research of Kyoto Prefectural University of Medicine (Authorization No. M2019‐516). We injected 5 × 10^6^ luciferase‐positive Rh30 cells into the right gastrocnemius muscle of the mice. After confirming tumor engraftment, the mice were randomly divided into two groups: one was fed a normal chow diet (NCD; fat content 12.0%/kcal) and the other was fed a low‐fat diet (LFD; fat content 1.3%/kcal). These diets were prepared by CLAIR Japan Inc. (Tokyo, Japan). Animals received food and sterile water ad libitum. Food intake was measured during the experiments. Tumor growth was monitored with in vivo bioluminescence imaging. The mice were intraperitoneally injected with D‐luciferin (150 mg/kg) 10 min before imaging and then anesthetized with 2% isoflurane during imaging. Images were captured with an IVIS Lumina Series III (PerkinElmer, Inc., Waltham, MA, USA) and Living Image v.2 (PerkinElmer, Inc.) was used to quantify regions of interest (ROIs) on the displayed images in photons per second (ph/s). These animal experiments were performed in accordance with the Animal Research Reporting In Vivo experiments (ARRIVE) guidelines.

### 2.3 Inhibitory reagents

The malonyl‐CoA decarboxylase inhibitor MCDi (CBM‐3001106) was kindly provided by Gary D. Lopaschuk.^20^, ^21^ Bafilomycin A1 (Baf A1) was purchased from Cayman Chemical (Ann Arbor, MI, USA). These reagents were dissolved in DMSO. In all experiments, the percentage of DMSO was less than 0.1%.

### 2.4 WST‐8 cell viability assay

To assess cell viability, WST‐8 colorimetric assays were performed using the Cell Counting Kit‐8 (Nacalai Tesque, Kyoto, Japan) according to the manufacturer's protocol. The cells were seeded in 96‐well plates in 100 µl culture medium for 24 h and the necessary reagents were added. Cell viability was determined by measuring the optical density (OD) at 450 nm with a microplate reader (Multiskan^TM^ JX; Thermo Labsystems, Santa Rosa, CA, USA).

### 2.5 Cell cycle analysis

To examine the effects of MCDi on the cell cycle, we cultured RMS cells with MCDi (10 µM) or DMSO for 24 h. The cells were then isolated by scraping, washed with PBS, and incubated at room temperature (22–25℃) with propidium iodide for 30 min to stain the DNA. We determined DNA content using a FACS Calibur flow cytometer (BD Biosciences, San Jose, CA, USA) and analyzed the status of the cell cycle using FlowJo v.9 (FlowJo LLC, Ashland, OR, USA), as described previously.^1^

### 2.6 Apoptosis analysis

We analyzed cell death after Annexin V‐FITC/propidium iodide staining using a TACS annexin‐V apoptosis detection kit (R&D BioSystems, Minneapolis, MN, USA) according to the manufacturer's instructions. Data were analyzed with FlowJo.

### 2.7 Quantitative real‐time PCR (qRT‐PCR)

To determine the mRNA expression levels, we extracted total RNA from RMS cells and tumor tissues were removed from xenograft models using a NucleoSpin RNA II kit (Macherey‐Nagel, Duren, Germany). cDNA was synthesized using ReverTra Ace qPCR RT Master Mix (Toyobo, Osaka, Japan). We prepared the working solution for qRT‐PCR with TB Green Premix Ex Taq II (Tli RNaseH Plus) (Takara, Shiga, Japan) and analyzed it using the AB 7500 Real‐Time PCR System (Applied Biosystems, Tokyo, Japan). The PCR steps were as follows: initial denaturation at 95℃ for 30 s, followed by 40 cycles of 95℃ for 5 s, and 60℃ for 34 s. The PCR primer sequences are shown in Table S1. *β*‐*actin* was used as the internal standard.

### 2.8 Western blot

RMS cells were administered MCDi or DMSO and incubated for 24 h. Cells were then lysed with RIPA lysis buffer (Nacalai Tesque). The products obtained by homogenization were centrifuged at 10,000×*g* for 5 min at 4℃ and the supernatants were collected. Protein concentrations were measured with a Bio‐Rad protein assay kit (Bio‐Rad, Tokyo, Japan). The proteins were electrophoresed on 4%–20% sodium dodecyl sulfate polyacrylamide gels followed by transferring to PVDF membranes and blocking with Blocking One (Nacalai Tesque). Can Get Signal solutions 1 and 2 (Toyobo) were used to diluted the primary and secondary antibodies, respectively. The membrane was incubated with primary antibodies against total AMP‐activated protein kinase (AMPKα) (1:1000), phospho‐AMPKα (Thr172) (1:1000), tuberous sclerosis 2 (TSC2) (1:1000), phospho‐TSC2 (1:1000), p70S6kinase (p70S6K) (1:1000), phospho‐p70S6K (1:1000), Waf‐p21 (p21) (1:1000), LC3A/B (1:1000), and β‐actin (1:5000). All abovementioned primary antibodies were purchased from Cell Signaling Technology (CST; Danvers, MA, USA). The secondary antibody was dissolved with HRP‐conjugated donkey anti‐rabbit immunoglobulin G (IgG) to 1:10,000 (for AMPK, p‐AMPK, TSC2, p‐TSC2, p70S6K, p‐p70S6K, p21, LC3A/B) (GE Healthcare, Tokyo, Japan) or HRP‐conjugated sheep anti‐mouse IgG (for β‐actin) (GE Healthcare). Antibody binding was measured using an enhanced HRP‐luminol chemiluminescence system. Raw western blot data are represented in Figure S1.

### 2.9 Immunofluorescence microscopy

Cells were cultured on Falcon 8‐well Culture Slides (354118, BD Falcon, Corning, Inc.), then fixed with 4% paraformaldehyde for 20 min at room temperature, permeabilized with 0.1% Triton X‐100, washed with PBS, and blocked with Blocking One Histo (Nacalai Tesque). Then, they were incubated with anti‐LC3A/B antibody (1:100) overnight at 4℃ then with Alexa Fluor 488‐conjugated goat anti‐rabbit IgG (H+L) cross‐absorbed secondary antibody (A‐11008, 1:200, Life Technologies, Tokyo, Japan) for 2 h at with shading. Nuclei were stained with HardSet Antifade Mounting Medium with DAPI (H‐1500, Vector Laboratories) for 15 min. Slides were imaged with a KEYENCE BZ‐X710 fluorescence microscope (Keyence Corp.).

### 2.10 Immunohistochemistry

Immunohistochemical staining was performed as described previously.^22^ Briefly, tumors removed from the xenografted mice were fixed with 4% paraformaldehyde, embedded in paraffin, sliced to a thickness of 4 µm, and placed on glass slides. After deparaffinization and rehydration, tissue antigens were retrieved with citrate buffer (pH 6), and endogenous peroxidase activity was inhibited by incubating the tissue sections with 3% H_2_O_2_ for 10 min. The sections were then blocked with Blocking One Histo (Nacalai Tesque) and incubated with mouse anti‐Ki67 antibody (M7240, DAKO, Glostrup, Denmark) (1:100) for 20 min at room temperature. Incubation with the secondary antibody (HRP‐conjugated goat anti‐mouse IgG antibody; ab214879, pre‐diluted, Abcam, Cambridge, UK) was performed for 30 min. Hematoxylin (8656, Sakura Finetek, Tokyo, Japan) was used for counterstaining the nucleus. The signals were detected by DAB (K3468, DAKO).

### 2.11 Metabolomics analysis

We performed metabolomic analysis using C‐Scope (Human Metabolome Technologies), according to the recommended protocol.^23^ Rh30 cells administered with DMSO or MCDi for 24 h were used as samples. In brief, after washing twice with 5% mannitol solution, 800 µl methanol, and 550 µl of 8 µM internal standard were added to the cells. Next, the cells were centrifuged at 4℃, 2300 × *g* for 5 min. The supernatants were then collected by centrifugation through a 5 kDa‐cutoff filter at 4℃, 8100 × *g* for 3 h. The concentrations of all charged compounds were measured by capillary electrophoresis time‐of‐flight mass spectrometry (CE‐TOFMS) and capillary electrophoresis tandem mass spectrometry (CE‐QqQMS; CE‐MS/MS) according to a previously described method.^24^

### 2.12 Statistical analysis

**FIGURE 1:**
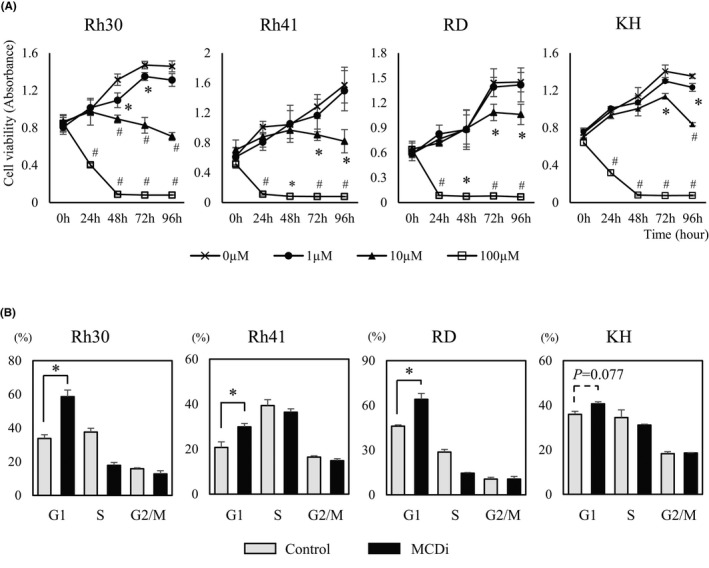
Inhibition of lipid metabolism in RMS cells induces suppression of cell proliferation and cell cycle arrest. (A) Growth curves of RMS cells over 96 h after administration of MCDi. Curves generated by WST8 assay. (B) Flow cytometry assay to calculate the percentage of cells present in each phase of the cell cycle at 24 h after drug administration (control (DMSO) vs. MCDi 10 µM). All results are shown as mean ± SD of three independent experiments. (*, *p* < 0.05 vs. control; #, *p* < 0.01 vs. control)

**FIGURE 2:**
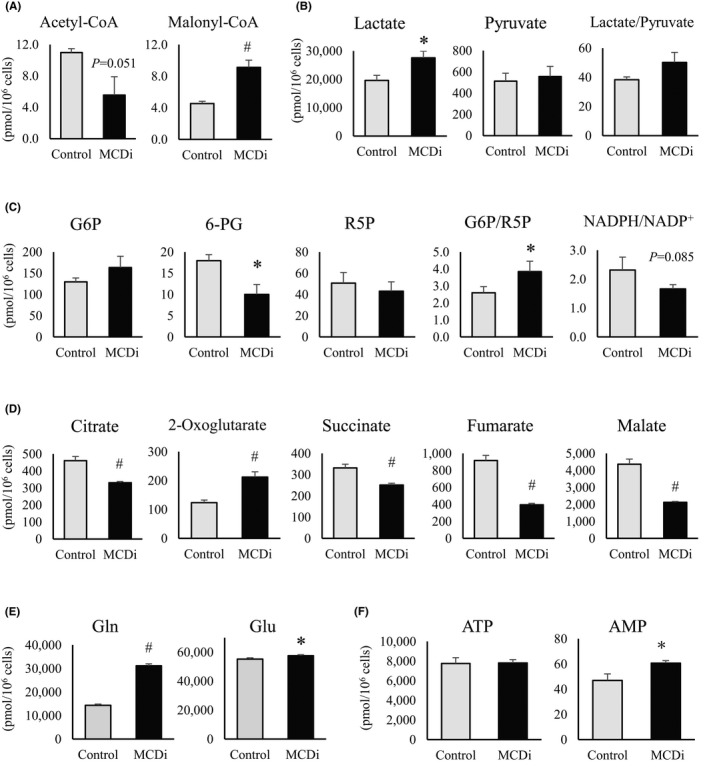
Metabolomic analysis confirms that inhibition of lipid metabolism altered cancer‐specific energy metabolism in Rh30 cell. Comparison of intermediate metabolites at 24 h after drug administration (DMSO vs. MCDi 10 µM); (A) acetyl‐CoA and malonyl‐CoA, (B) lactate, pyruvate, and the ratio of lactate/pyruvate, (C) glucose‐6‐phosphate (G6P), 6‐phosphogluconate (6‐PG), ribose‐5‐phosphate (R5P), and G6P/R5P,and NADPH/NADP ratios, (D) citrate, 2‐oxoglutarate, succinate, fumarate and malate, (E) glutamine (Gln), and glutamate (Glu), (F) ATP and AMP. All results are shown as mean ±SD of three independent experiments. (*, *p* < 0.05 vs. control; #, *p* < 0.01 vs. control)

**FIGURE 3:**
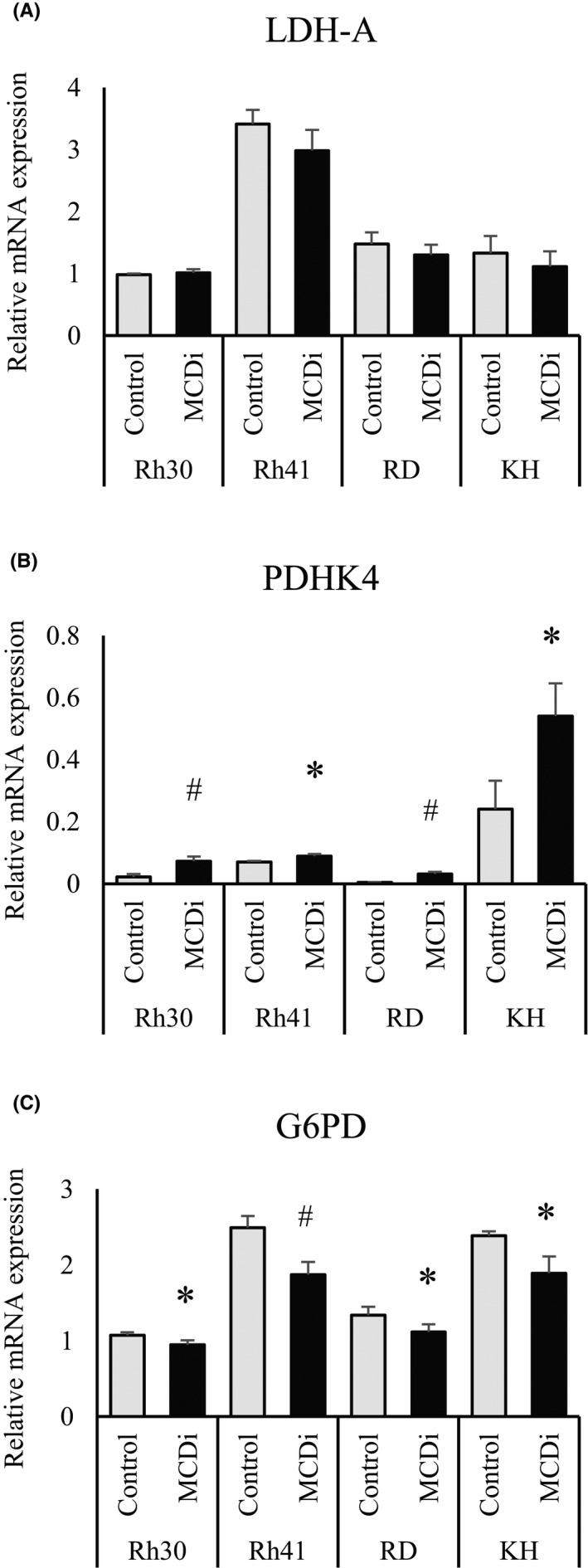
Suppression of lipid metabolism leads to inhibition of pyruvate influx into mitochondrial TCA cycle and inactivation of the PPP. Comparison of mRNA expression levels of (A) *LDHA*, (B) *PDHK4*, and (C) *G6PD* in RMS cell lines at 12 h after drug administration (DMSO vs. MCDi 10 µM). All results are shown as mean ±SD of four independent experiments. (*, *p* < 0.05 vs. control; #, *p* < 0.01 vs. control)

All data are presented as the mean ±standard deviation (SD). To compare the means between groups, we used a two‐tailed *t*‐test in Figures 1, 2, 3 and 5 (*n* = 3 or 4). Mann–Whitney's *U*‐test was performed for the analysis in Figure 6 (*n* = 5 in each group). Differences with a *p*‐value <0.05 were considered statistically significant.

## 3 RESULTS

### 3.1 Inhibition of lipid metabolism by MCDi suppressed cell proliferation and induced cell cycle arrest in RMS cells

We first investigated the effect on tumor growth suppressing lipid metabolism with the MCDi.^20^, ^21^ Administration of MDCi to the ARMS cell lines Rh30 and Rh41, and the ERMS cell lines RD and KH, resulted in concentration‐ and time‐dependent inhibition of growth (Figure 1A). Notably, the mRNA expression level of carnitine palmitoyltransferase (CPT) 1 isozymes, the target of malonyl‐CoA, was different in each cell line (Figure S2). It has been reported that *PAX3*‐*FKHR* (*PAX3*‐*FOXO1*), a fusion gene characteristic of ARMS, is involved in the transcription of *CPT1A* and regulates tumor cell infiltration and metastasis.^25^ MCDi administration increases the level of malonyl‐CoA, which inhibits CPT1 and reduces fatty acid uptake in mitochondria.^26^ In this study, the inhibitory effect of MCDi on tumor growth was similar for both ARMS and ERMS. Furthermore, the effect of inhibiting lipid metabolism on the cell cycle was examined. Administration of 10 µM MCDi‐induced G1 cell cycle arrest in Rh30, Rh41, and RD with statistically significant differences. In case of KH, there was no significant difference, although the G1 phase cell population tended to increase (*p *= 0.077) (Figure 1B).

### 3.2 Metabolomic analysis revealed that inhibition of lipid metabolism affects tumor‐specific energy metabolism in RMS cells

We performed a metabolomic analysis of Rh30 cells treated for 24 h with DMSO or 10 µM MCDi. Reflecting the action of MCDi, a significant increase in malonyl‐CoA levels and decrease in acetyl‐CoA levels were observed (Figure 2A). The promotion of lactic acid synthesis was confirmed (Figure 2B). Moreover, a markedly decreased level of 6‐phosphogluconate (6‐PG) along with a declining NADPH/NADP^+^ ratio (*p* = 0.085) and a significantly increased 6‐PG/ribose 5‐phosphate (R5P) ratio indicated inactivation of the PPP (Figure 2C). The levels of TCA cycle intermediate metabolites, except for 2‐oxoglutaric acid (2‐OG), were significantly reduced by the suppression of lipid metabolism (Figure 2D). Both glutamine (Gln) and glutamate (Glu) levels were significantly increased by the administration of MCDi (Figure 2E), suggesting that the increase in 2‐OG was a result of the replacement pathway for glutamine degradation. There was no difference in the amount of ATP, although AMP levels were significantly increased in MCDi‐treated cells (Figure 2F).

Next, we compared the mRNA expression levels of key enzymes located at the junction of glycolysis to determine whether the changes in these metabolites due to MCDi administration occurred similarly in other RMS cell lines. Lactate dehydrogenase A (LDH‐A), an enzyme that converts pyruvate to lactate, did not show a significant change (Figure 3A); however, the expression of pyruvate dehydrogenase (PDH) kinase (PDHK4), which inhibits PDH conversion of pyruvate to acetyl‐CoA, was significantly increased in all cell lines due to the inhibition of lipid metabolism (Figure 3B). In addition, the mRNA expression of glucose 6‐phosphate dehydrogenase (G6PD), the first rate‐limiting enzyme in the PPP which converts glucose 6‐phosphate (G6P) to 6‐phosphogluconic acid (6PG), was significantly reduced (Figure 3C). These results indirectly demonstrate that the suppression of lipid metabolism by MCDi administration caused similar metabolic changes in all four RMS cell lines.

### 3.3 Inhibition of lipid metabolism promotes autophagy signaling via the AMPK‐mTOR pathway and increases p21 expression

**FIGURE 4:**
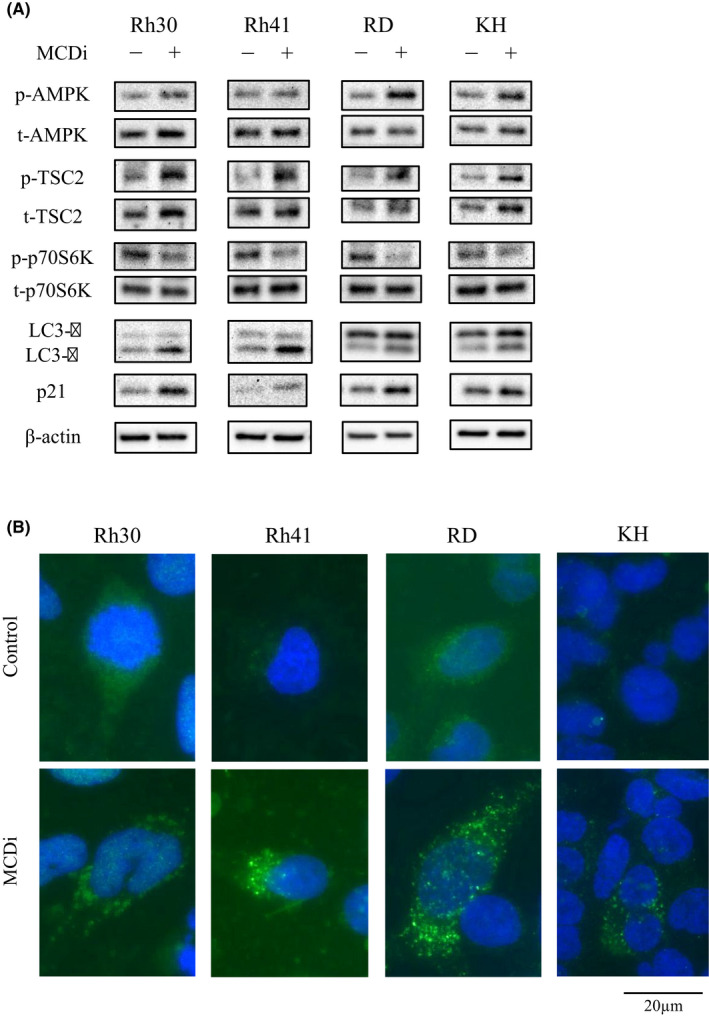
Inhibition of lipid metabolism induces autophagy signaling through increased phosphorylation of AMPK, and increased p21 protein expression. (A) Protein expression from RMS cells at 24 h after drug administration (DMSO vs. MCDi 10 µM). β‐actin was used as the internal control. (B) RMS cells were treated with DMSO or MCDi (10 µM, 48 h), followed by immunofluorescence staining for‐LC3A/B. Green spots represent autophagosome formation (LC3 puncta). Nuclei were labeled with DAPI

Western blot analyses were performed to assess the protein expression level of AMPK, which is an important intracellular energy sensor. After the administration of DMSO or 10 µM MCDi for 24 h, the phosphorylation of AMPK was increased (Figure 4A), as were AMP levels (Figure 2F), which activate AMPK. Moreover, inhibition of lipid metabolism increased the phosphorylation level of TSC2 located downstream of AMPK. Further, the phosphorylation of p70S6K, located downstream of mTORC1, was reduced. Expression of p21, a cell cycle suppressor, was also increased. Moreover, LC3A/B‐II protein levels were increased after MCDi administration (Figure 4A). Usually, when autophagy is promoted, LC3‐I is converted into its phosphatidylethanolamine‐conjugated form, LC3‐II, which is a key factor in the maturation of autophagosome. Immunofluorescence staining revealed that inhibition of lipid metabolism increased LC3 puncta in the cytoplasm of RMS cells, indicating the formation of autophagosomes (Figure 4B).

### 3.4 Inhibiting both lipid metabolism and autophagy suppresses tumor cell proliferation with increased apoptotic cell death

**FIGURE 5:**
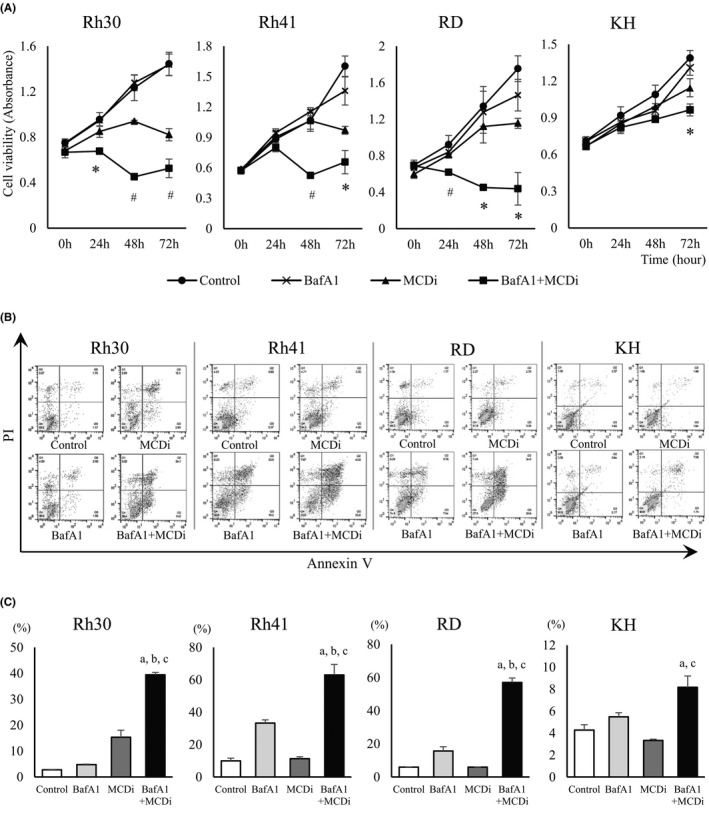
Inhibition of both lipid metabolism and autophagy suppressed the growth of RMS cell lines through increased apoptosis. (A) Cell viability assessed by WST8 assay. RMS cell lines were treated with DMSO, BafA1 (2.5 nM), and/or MCDi (10 µM) for 72 h. Data are shown as mean ±SD of three independent experiments. (*, *p* < 0.05 vs. control; #, *p* < 0.01 vs. control.) (B) Apoptosis assay. RMS cell lines were treated with DMSO, BafA1 (2.5 nM), and/or MCDi (10 µM) for 48 h. Dot plots showed the distribution of cell populations. (C) The proportion of apoptotic cells assessed by Annexin V‐FITC and propidium iodide (PI) staining. The results are shown as mean ±SD of three independent experiments. (a, *p* < 0.05 vs. control; b, *p* < 0.05 vs. BafA1; c, *p* < 0.05 vs. MCDi)

Then we investigated whether the autophagy induced by the inhibition of lipid metabolism was a tumor‐protective response or the cytotoxic event known as autophagic cell death. BafA1 inhibits the late phase autophagy by preventing the maturation of autophagic vacuoles through the inhibition of fusion between autophagosomes and lysosomes. When MCDi and BafA1 were used together, cell proliferation was suppressed to a greater degree in combination group than individually (Figure 5A). Analysis revealed that the annexin V‐positive cell population increased following concomitant administration, suggesting further induction of apoptotic cell death (Figure 5B and C).

### 3.5 A fat‐restricted diet suppresses RMS growth in an orthotopic xenograft mouse model through decreasing lipid metabolism

**FIGURE 6:**
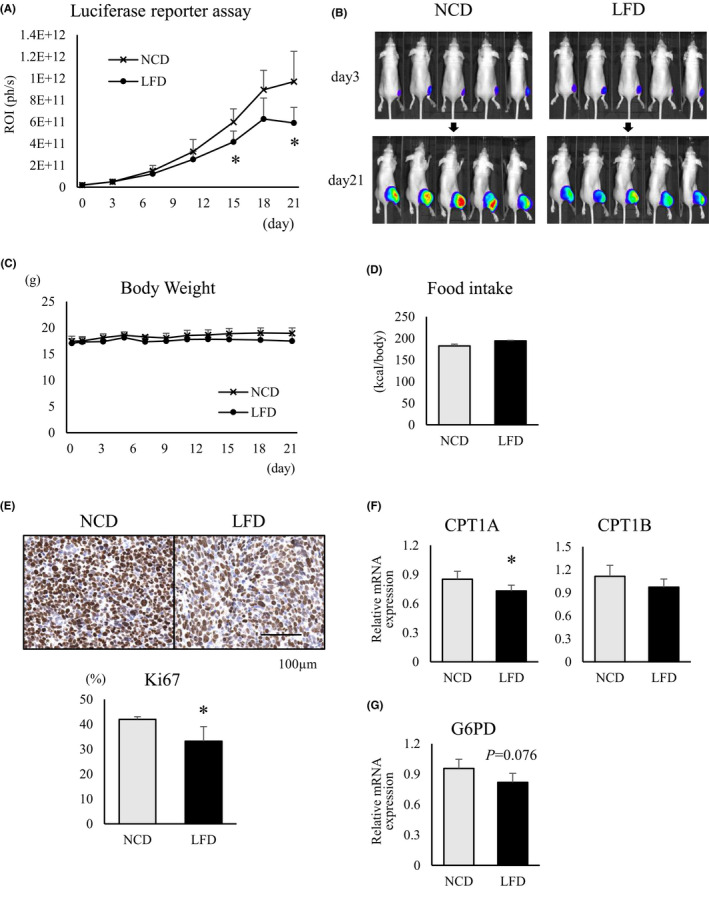
In an orthotopic xenograft model, LFD showed growth inhibitory effect against RMS through reduced lipid metabolism. (A) Ten mice transplanted with luciferase‐positive Rh30 cells were divided into two groups (*n* = 5 each) after confirming tumor engraftment. After starting NCD or LFD, tumor growth was quantified using bioluminescence imaging. (B) Comparison of bioluminescence images on 3 and 21 days after the start of the diets. (C) Changes in body weight. (D) Comparison of caloric intake. (E) Tissue sections of tumor samples prepared from mice sacrificed on day 21 were immunohistochemically stained for anti‐Ki67. Upper panels, representative stained images of both. Lower panel, graph of Ki67‐positive rate. mRNA expression levels of (F) *CPT1* isozymes and (G) *G6PD* in tumor samples from each group on day 14. All results are shown as mean ±SD, *n* = 5 for each group. (*, *p* < 0.05 vs. NCD‐fed group)

After confirming the engraftment of transplanted luciferase‐positive Rh30 cells in a xenograft mouse model, the bioluminescence of the tumor cells was evaluated over a 3‐ week period after initiation of NCD or LFD feeding. LFD‐fed mice had lower tumor‐related bioluminescence intensities than NCD‐fed mice (Figure 6A). At 21 days after diet change, the tumor sizes of LFD‐fed mice were smaller than those of NCD‐fed mice (Figure 6B). During the observation period, there was no significant difference in body weight and calorie intake between the NCD‐ and LFD‐fed mice (Figure 6C and D). Mice were euthanized to excise the tumor mass and perform IHC staining for Ki67. The Ki67‐positive rate was significantly lower in tumor samples from LFD‐fed mice than from NCD‐fed mice (Figure 6E). Moreover, comparison of the mRNA expression levels of CPT1 isozymes, which are rate‐limiting enzymes for lipid metabolism in mitochondria, showed that in LFD‐fed mice, expression of *CPT1A* mRNA was significantly lower than in tumors from NCD‐fed mice, and *CPT1B* expression tended to decrease as well (Figure 6F). In addition, *G6PD* mRNA expression was relatively reduced in LFD‐fed mice compared to NCD‐fed mice (*p *= 0.076) (Figure 6G).

## 4 DISCUSSION

Therapeutic approaches that specifically target tumors and have few systemic side effects are important for cancer treatment. In recent years, many researchers have focused on therapeutic strategies targeting tumor‐specific energy metabolism. However, there are few studies on the metabolism of childhood cancers, especially RMS. The present study yields novel findings concerning a therapeutic strategy for RMS, focusing on lipid metabolism.

The Warburg effect has been previously reported to occur in RMS.^10^, ^12^ The use of stable isotopes to analyze intracellular metabolism revealed that glucose uptake was significantly increased and lactate synthesis was enhanced in the Rh30 cell line compared to normal myocytes, demonstrating upregulated glycolysis in RMS.^10^ Moreover, the mitochondrial TCA cycle was upregulated in Rh30, indicating that energy sources other than glucose, such as glutamic acid and fatty acids, were also essential for the TCA cycle. Thus, we hypothesized that suppressing lipid metabolism in RMS would upset the balance of tumor‐specific energy metabolism, or the Warburg effect, leading to an antitumor effect.

Metabolomic analysis revealed that most intermediates in the mitochondrial TCA cycle were significantly reduced by the administration of MCDi, as would be expected if fatty acid oxidation was inhibited. 2‐OG was the only TCA cycle intermediate metabolite that showed increased levels, likely owing to the anaplerotic pathway, which takes up glutamate from the cytoplasm into the mitochondria.^27^ Metabolomic analysis further revealed that the PPP was downregulated and nucleic acid synthesis was suppressed in MCDi‐treated RMS. AMPK is a key nutrient sensor that controls various intracellular signals by reflecting the nutritional status in both normal and tumor cells.^28^ Recently, AMPK has been reported to suppress the expression of G6PD, the rate‐limiting enzyme in the first stage of PPP, by inactivating the transcriptional activities of cyclic AMP‐response element‐binding protein (CREB) and CREB‐regulated transcriptional coactivator‐1 (CRTC‐1).^29^, ^30^ In our study, the inhibition of lipid metabolism in RMS cell lines increased AMPK phosphorylation, reflecting the intracellular low‐energy state, and suppressed the expression of *G6PD* mRNA. This suggests that the inhibition of lipid metabolism suppressed the PPP in RMS cells via the AMPK‐CRCT1‐CREB‐G6PD pathway. Inhibition of lipid metabolism‐induced cell cycle arrest in the G1 phase and increased the expression of p21 in all RMS cell lines. p21 is a major target of p53 activity and is thus associated with cell cycle arrest. In RMS cells, especially Rh30, Rh41, and RD cell lines, the tumor suppressor protein p53 is either mutated or deleted, and therefore its function is suppressed.^31^, ^32^, ^33^ Inhibition of lipid metabolism suppressed the PPP in RMS cells, which might induce G1 arrest by promoting p21 expression via a p53‐independent pathway.^34^, ^35^ Overall, we propose that the imbalanced tumor‐specific energy metabolism due to the suppression of lipid metabolism leads to an antitumor effect in RMS cells.

Autophagy facilitates the production of energy substrates required for cells to maintain their survival during starvation and breaks down the waste products accumulated inside cells, such as abnormal proteins and organelles with reduced function.^36^, ^37^ While various signals control autophagy, AMPK is a major factor that induces autophagy, especially under nutrient deprivation.^38^ mTORC1 is a major suppressor of autophagy signaling.^39^ As mentioned above, AMPK inhibits mTORC1 through the activation of TSC2, and also directly activates the unc‐51‐like autophagy activating kinase (Ulk1), followed by the induction of autophagy signals.^40^, ^41^ Autophagy is present not only in normal cells but also in cancer cells.^37^, ^42^ In this study, the suppression of lipid metabolism increased AMPK phosphorylation in RMS cell lines, resulting in activation of the autophagy pathway. Additionally, western blotting and immunofluorescence staining confirmed increased expression of LC3‐II, which is a component of the autophagosome membrane and is therefore considered an autophagy marker.

Previous studies have shown that the role of autophagy in tumors, whether beneficial or harmful, is complex and depends on the type of cancer. The advantage of autophagy in cancer cells is its protective role against various environmental stressors, and blocking this compensatory response increases cancer cell death.^43^, ^44^ However, activation of autophagy itself induces autophagic cell death in cancer cells.^45^ This type of programmed cell death is defined by reduced cancer cell death due to inhibition of autophagy. It has been reported that several therapeutic approaches induced autophagy in RMS. In addition, when the increased autophagy was suppressed, apoptotic cell death was induced.^46^, ^47^ Consistent with these previous studies, we found that suppression of lipid metabolism in RMS‐induced autophagy. Moreover, inhibition of autophagy led to promotion of apoptosis. Therefore, autophagy plays a tumor‐protective role in RMS, and combination therapy by suppressing both lipid metabolism and autophagy may be promising as a therapeutic strategy.

Based on the results of in vitro experiments, we examined the lipid dependence of RMS in orthotopic xenograft models by restricting dietary exogenous fats. Comparison between NCD‐ and LFD‐fed mice showed no significant difference in body weight and total calorie intake during the observation period. However, bioluminescence imaging showed significantly suppressed tumor growth in LFD‐fed mice compared to NCD‐fed mice. Since calorie restriction was not involved, the difference in exogenous lipid uptake between the two groups was considered the cause of this difference. Moreover, *CPT1A* mRNA expression was significantly decreased in the tumors of LFD‐fed mice, suggesting that not only the uptake of exogenous lipids, but also the import of fatty acids, into mitochondria were reduced. A significant decrease in the expression of Ki67, a cell cycle‐related protein, was observed in LFD‐fed mice, indicating that the reduced lipid metabolism led to suppression of the cell cycle. PPP might also have been affected, as the expression of *G6PD* mRNA tended to decrease. Thus, our in vivo experiments showed that the reduced exogenous lipid supply due to LFD‐affected lipid metabolism in RMS and exerted a tumor growth inhibitory effect.

Recently, there have been reports that a high‐fat diet (HFD) promotes the growth of several types of tumors. In colorectal cancer, an increased level of HFD‐derived palmitic acid induced the activation of hormone‐sensitive lipase through the upregulation of cAMP/PKA signaling, which was promoted by increasing β2 adrenergic receptor expression, enhancing the supply of fatty acids as an energy substrate in tumor cells; this affected the metabolic phenotype and made the tumor more malignant.^48^ HFD also increased the population of myeloid‐derived suppressor cells, which have been reported to be facilitators of tumor growth, resulting in the stimulation of inflammation in the tumor microenvironment and affecting the malignancy of the tumor.^49^, ^50^ In other words, the availability of a large amount of fatty acids contributes to tumor growth in various ways. Like normal cells, cancer cells have lipid droplets in their cytoplasm, which enable them to adjust to adverse conditions such as starvation and oxidative stress.^51^ Furthermore, many lipid metabolism‐related enzymes are associated with undesirable characteristics of cancer cells, such as invasion and metastasis.^52^ High lipid availability is advantageous for tumor cells, whereas low lipid content may be disadvantageous for cancer growth; however, there have been few reports indicating that the suppression of lipid utilization exerts an antitumor effect.

This study included certain limitations. In the in vitro experiments, we only compared the effects of using a drug that suppresses intracellular lipid metabolism, and did not compare the effects on inhibiting the utilization of the exogeneous lipids, such as by reducing the fat content in the medium. The in vivo experiments in this study were conducted in mice, and the effects of lipid metabolism inhibition on humans need to be assessed. Further studies are warranted to identify the master key regulator that plays a central role in mediating the changes in the metabolic balance, in response to the suppression of lipid metabolism.

In conclusion, the present study revealed that inhibition of lipid metabolism suppressed the PPP in RMS. As a result, the synthesis of nucleic acids essential for the growth of cancer cells and antioxidants required for maintaining oxidative balance decreased, leading to inhibition of tumor growth inhibitory due to cell cycle arrest. It has been suggested that AMPK might play a key role in this effect. Autophagy signaling induced by the AMPK‐mTOR pathway has been thought to be a tumor‐protective response in RMS. This is because apoptotic cell death was induced via inhibition of autophagy, which was promoted by suppressing lipid metabolism. Including the experimental results in mouse xenograft models, we report for the first time that RMS relies on lipid metabolism to maintain its growth. Therefore, we believe that reducing lipid intake is a promising therapeutic strategy of RMS.

## CONFLICT OF INTERESTS

The authors have no conflict of interests.

## ETHICAL APPROVAL STATEMENT

Our animal experiments were approved by the Committee for Animal Research of Kyoto Prefectural University of Medicine (Authorization No. M2019‐516), and performed in accordance with the Animal Research Reporting In Vivo experiments (ARRIVE) guidelines.

## Supporting information

**Figure S1**.
**Figure S2**.

Table S1.

## Data Availability

The data that support the findings of this study are available from the corresponding author, KK, upon reasonable request.

## References

[1] Kikuchi K, Hettmer S, Aslam MI, et al. Cell‐cycle dependent expression of a translocation‐mediated fusion oncogene mediates checkpoint adaptation in rhabdomyosarcoma. PLoS Genet. 2014;10(1):e1004107. 10.1371/journal.pgen.1004107 24453992 PMC3894165

[2] Otabe O, Kikuchi K, Tsuchiya K, et al. MET/ERK2 pathway regulates the motility of human alveolar rhabdomyosarcoma cells. Oncol Rep. 2017;37(1):98‐104. 10.3892/or.2016.5213 27840956

[3] Hayes‐Jordan A, Andrassy R. Rhabdomyosarcoma in children. Curr Opin Pediatr. 2009;21(3):373‐378. 10.1097/MOP.0b013e32832b4171 19448544

[4] Xia SJ, Pressey JG, Barr FG. Molecular pathogenesis of rhabdomyosarcoma. Cancer Biol Ther. 2002;1(2):97‐104. 10.4161/cbt.51 12170781

[5] Panda SP, Chinnaswamy G, Vora T, et al. Diagnosis and management of rhabdomyosarcoma in children and adolescents: ICMR consensus document. Indian J Pediatr. 2017;84(5):393‐402. 10.1007/s12098-017-2315-3 28378141

[6] Warburg O. On the origin of cancer cells. Science. 1956;123(3191):309‐314. 10.1126/science.123.3191.309 13298683

[7] De Preter G, Neveu MA, Danhier P, et al. Inhibition of the pentose phosphate pathway by dichloroacetate unravels a missing link between aerobic glycolysis and cancer cell proliferation. Oncotarget. 2016;7(3):2910‐2920. 10.18632/oncotarget.6272 26543237 PMC4823080

[8] Patra KC, Hay N. The pentose phosphate pathway and cancer. Trends Biochem Sci. 2014;39(8):347‐354. 10.1016/j.tibs.2014.06.005 25037503 PMC4329227

[9] Jiang P, Du W, Wu M. Regulation of the pentose phosphate pathway in cancer. Protein Cell. 2014;5(8):592‐602. 10.1007/s13238-014-0082-8 25015087 PMC4112277

[10] Fan TWM, Kucia M, Jankowski K, et al. Rhabdomyosarcoma cells show an energy producing anabolic metabolic phenotype compared with primary myocytes. Mol Cancer. 2008;7:79. 10.1186/1476-4598-7-79 18939998 PMC2577687

[11] Noguchi T, Inoue H, Tanaka T. The M1‐ and M2‐type isozymes of rat pyruvate kinase are produced from the same gene by alternative RNA splicing. J Biol Chem. 1986;261(29):13807‐13812. 3020052

[12] Sugito N, Taniguchi K, Kuranaga Y, et al. Cancer‐specific energy metabolism in rhabdomyosarcoma cells is regulated by microRNA. Nucleic Acid Ther. 2017;27(6):365‐377. 10.1089/nat.2017.0673 28981396

[13] Deberardinis RJ, Sayed N, Ditsworth D, Thompson CB. Brick by brick: metabolism and tumor cell growth. Curr Opin Genet Dev. 2008;18(1):54‐61. 10.1016/j.gde.2008.02.003 18387799 PMC2476215

[14] Menendez JA. Fine‐tuning the lipogenic/lipolytic balance to optimize the metabolic requirements of cancer cell growth: molecular mechanisms and therapeutic perspectives. Biochim Biophys Acta. 2010;1801(3):381‐391. 10.1016/j.bbalip.2009.09.005 19782152

[15] Douglass EC, Valentine M, Etcubanas E, et al. A specific chromosomal abnormality in rhabdomyosarcoma. Cytogenet Cell Genet. 1987;45(3–4):148‐155. 10.1159/000132446 3691179

[16] Petak I, Douglas L, Tillman DM, et al. Pediatric rhabdomyosarcoma cell lines are resistant to Fas‐induced apoptosis and highly sensitive to TRAIL‐induced apoptosis. Clin Cancer Res. 2000;6(10):4119‐4127. 11051265

[17] Sugimoto T, Kuroda H, Kuwahara Y, et al. Cellular and molecular characteristics of established childhood soft‐tissue sarcoma cell lines. J Cancer Res Ther Oncol. 2019;7(202):1‐12.

[18] Tsuchiya K, Hosoi H, Misawa‐Furihata A, et al. Insulin‐like growth factor‐I has different effects on myogenin induction and cell cycle progression in human alveolar and embryonal rhabdomyosarcoma cells. Int J Oncol. 2007;31(1):41‐47. 17549403

[19] Nakagawa N, Kikuchi K, Yagyu S, et al. Mutations in the RAS pathway as potential precision medicine targets in treatment of rhabdomyosarcoma. Biochem Biophys Res Commun. 2019;512(3):524‐530. 10.1016/j.bbrc.2019.03.038 30904164

[20] Samokhvalov V, Ussher JR, Fillmore N, et al. Inhibition of malonyl‐CoA decarboxylase reduces the inflammatory response associated with insulin resistance. Am J Physiol Endocrinol Metab. 2012;303(12):E1459‐E1468. 10.1152/ajpendo.00018.2012 23074239

[21] Ussher JR, Fillmore N, Keung W, et al. Genetic and pharmacological inhibition of malonyl CoA decarboxylase does not exacerbate age‐related insulin resistance in mice. Diabetes. 2016;65(7):1883‐1891. 10.2337/db15-1145 27207536

[22] Cao D, Kishida S, Huang P, et al. A new tumorsphere culture condition restores potentials of self‐renewal and metastasis of primary neuroblastoma in a mouse neuroblastoma model. PLoS One. 2014;9(1):e86813. 10.1371/journal.pone.0086813 24466252 PMC3899333

[23] Makinoshima H, Takita M, Matsumoto S, et al. Epidermal growth factor receptor (EGFR) signaling regulates global metabolic pathways in EGFR‐mutated lung adenocarcinoma. J Biol Chem. 2014;289(30):20813‐20823. 10.1074/jbc.M114.575464 24928511 PMC4110289

[24] Soga T, Baran R, Suematsu M, et al. Differential metabolomics reveals ophthalmic acid as an oxidative stress biomarker indicating hepatic glutathione consumption. J Biol Chem. 2006;281(24):16768‐16776. 10.1074/jbc.M601876200 16608839

[25] Liu L, Wang YD, Wu J, et al. Carnitine palmitoyltransferase 1A (CPT1A): a transcriptional target of PAX3‐FKHR and mediates PAX3‐FKHR‐dependent motility in alveolar rhabdomyosarcoma cells. BMC Cancer. 2012;12:154. 10.1186/1471-2407-12-154 22533991 PMC3453510

[26] Fillmore N, Lopaschuk GD. Malonyl CoA: a promising target for the treatment of cardiac disease. IUBMB Life. 2014;66(3):139‐146. 10.1002/iub.1253 24591219

[27] DeBerardinis RJ, Mancuso A, Daikhin E, et al. Beyond aerobic glycolysis: transformed cells can engage in glutamine metabolism that exceeds the requirement for protein and nucleotide synthesis. Proc Natl Acad Sci USA. 2007;104(49):19345‐19350. 10.1073/pnas.0709747104 18032601 PMC2148292

[28] Shaw RJ, Kosmatka M, Bardeesy N, et al. The tumor suppressor LKB1 kinase directly activates AMP‐activated kinase and regulates apoptosis in response to energy stress. Proc Natl Acad Sci USA. 2004;101(10):3329‐3335. 10.1073/pnas.0308061100 14985505 PMC373461

[29] Mair W, Morantte I, Rodrigues APC, et al. Lifespan extension induced by AMPK and calcineurin is mediated by CRTC‐1 and CREB. Nature. 2011;470(7334):404‐408. 10.1038/nature09706 21331044 PMC3098900

[30] Laliotis GP, Bizelis I, Argyrokastritis A, Rogdakis E. Cloning, characterization and computational analysis of the 5’ regulatory region of ovine glucose 6‐phosphate dehydrogenase gene. Comp Biochem Physiol B Biochem Mol Biol. 2007;147(4):627‐634. 10.1016/j.cbpb.2007.04.001 17493856

[31] Hinson ARR, Jones R, Crose LES, et al. Human rhabdomyosarcoma cell lines for rhabdomyosarcoma research: utility and pitfalls. Front Oncol. 2013;3:183. 10.3389/fonc.2013.00183 23882450 PMC3713458

[32] Miyachi M, Kakazu N, Yagyu S, et al. Restoration of p53 pathway by nutlin‐3 induces cell cycle arrest and apoptosis in human rhabdomyosarcoma cells. Clin Cancer Res. 2009;15(12):4077‐4084. 10.1158/1078-0432.CCR-08-2955 19509161

[33] Shern JF, Chen L, Chmielecki J, et al. Comprehensive genomic analysis of rhabdomyosarcoma reveals a landscape of alterations affecting a common genetic axis in fusion‐positive and fusion‐negative tumors. Cancer Discov. 2014;4(2):216‐231. 10.1158/2159-8290.CD-13-0639. 24436047 PMC4462130

[34] Mahyar‐Roemer M, Roemer K. p21 Waf1/Cip1 can protect human colon carcinoma cells against p53‐dependent and p53‐independent apoptosis induced by natural chemopreventive and therapeutic agents. Oncogene. 2001;20(26):3387‐3398. 10.1038/sj.onc.1204440 11423989

[35] Tinkum KL, White LS, Marpegan L, et al. Forkhead box O1 (FOXO1) protein, but not p53, contributes to robust induction of p21 expression in fasted mice. J Biol Chem. 2013;288(39):27999‐28008. 10.1074/jbc.M113.494328 23918930 PMC3784713

[36] Mizushima N, Yoshimori T, Ohsumi Y. The role of Atg proteins in autophagosome formation. Annu Rev Cell Dev Biol. 2011;27:107‐132. 10.1146/annurev-cellbio-092910-154005 21801009

[37] Kimmelman AC, White E. Autophagy and tumor metabolism. Cell Metab. 2017;25(5):1037‐1043. doi:10.1016/j.cmet.2017.04.004. 28467923 PMC5604466

[38] Kim J, Kundu M, Viollet B, Guan KL. AMPK and mTOR regulate autophagy through direct phosphorylation of Ulk1. Nat Cell Biol. 2011;13(2):132‐141. 10.1038/ncb2152 21258367 PMC3987946

[39] Thoreen CC, Kang SA, Chang JW, et al. An ATP‐competitive mammalian target of rapamycin inhibitor reveals rapamycin‐resistant functions of mTORC1. J Biol Chem. 2009;284(12):8023‐8032. 10.1074/jbc.M900301200 19150980 PMC2658096

[40] Mao K, Klionsky DJ. AMPK activates autophagy by phosphorylating ULK1. Circ Res. 2011;108(7):787‐788. 10.1161/RES.0b013e3182194c29 21454792 PMC3619191

[41] Mack HI, Zheng B, Asara JM, Thomas SM. AMPK‐dependent phosphorylation of ULK1 regulates ATG9 localization. Autophagy. 2012;8(8):1197‐1214. 10.4161/auto.20586 22932492 PMC3679237

[42] Amaravadi R, Kimmelman AC, White E. Recent insights into the function of autophagy in cancer. Genes Dev. 2016;30(17):1913‐1930. 10.1101/gad.287524.116 27664235 PMC5066235

[43] Feng H, Cheng X, Kuang J, et al. Apatinib‐induced protective autophagy and apoptosis through the AKT‐mTOR pathway in anaplastic thyroid cancer. Cell Death Dis. 2018;9(10):1030. 10.1038/s41419-018-1054-3 30301881 PMC6177436

[44] Ma R, Zhang Y, Wang W, et al. Inhibition of autophagy enhances the antitumour activity of tigecycline in multiple myeloma. J Cell Mol Med. 2018;22(12):5955‐5963. 10.1111/jcmm.13865 30247801 PMC6237591

[45] Yao ZQ, Zhang X, Zhen Y, et al. A novel small‐molecule activator of Sirtuin‐1 induces autophagic cell death/mitophagy as a potential therapeutic strategy in glioblastoma. Cell Death Dis. 2018;9(7):767. 10.1038/s41419-018-0799-z 29991742 PMC6039470

[46] Moghadam AR, da Silva Rosa SC, Samiei E, et al. Autophagy modulates temozolomide‐induced cell death in alveolar rhabdomyosarcoma cells. Cell Death Discov. 2018;4:52. 10.1038/s41420-018-0115-9 PMC6202374 30416757

[47] Wang C, Qu J, Yan S, et al. PFK15, a PFKFB3 antagonist, inhibits autophagy and proliferation in rhabdomyosarcoma cells. Int J Mol Med. 2018;42(1):359‐367. 10.3892/ijmm.2018.3599 29620138 PMC5979828

[48] Fatima S, Hu X, Huang C, et al. High‐fat diet feeding and palmitic acid increase CRC growth in beta2AR‐dependent manner. Cell Death Dis. 2019;10(10):711. 10.1038/s41419-019-1958-6 31558710 PMC6763436

[49] Hayashi T, Fujita K, Nojima S, et al. High‐fat diet‐induced inflammation accelerates prostate cancer growth via IL6 signaling. Clin Cancer Res. 2018;24(17):4309‐4318. 10.1158/1078-0432.CCR-18-0106 29776955

[50] Clements VK, Long T, Long R, et al. Frontline Science: high fat diet and leptin promote tumor progression by inducing myeloid‐derived suppressor cells. J Leukoc Biol. 2018;103(3):395‐407. 10.1002/JLB.4HI0517-210R 29345342 PMC7414791

[51] Petan T, Jarc E, Jusović M. Lipid droplets in cancer: guardians of fat in a stressful world. Molecules. 2018;23(8):1941. 10.3390/molecules23081941 PMC6222695 30081476

[52] Luo X, Cheng C, Tan Z, et al. Emerging roles of lipid metabolism in cancer metastasis. Mol Cancer. 2017;16(1):76. 10.1186/s12943-017-0646-3 28399876 PMC5387196

