# Supplementary figures and images for: Inhibition of lipid metabolism exerts antitumor effects on rhabdomyosarcoma
Source: Cancer Med. 2021 Sep 2;10(18):6442–55. doi: 10.1002/cam4.4185 (PMC8446407; doi:10.1002/cam4.4185)

**Figure S1(1).**

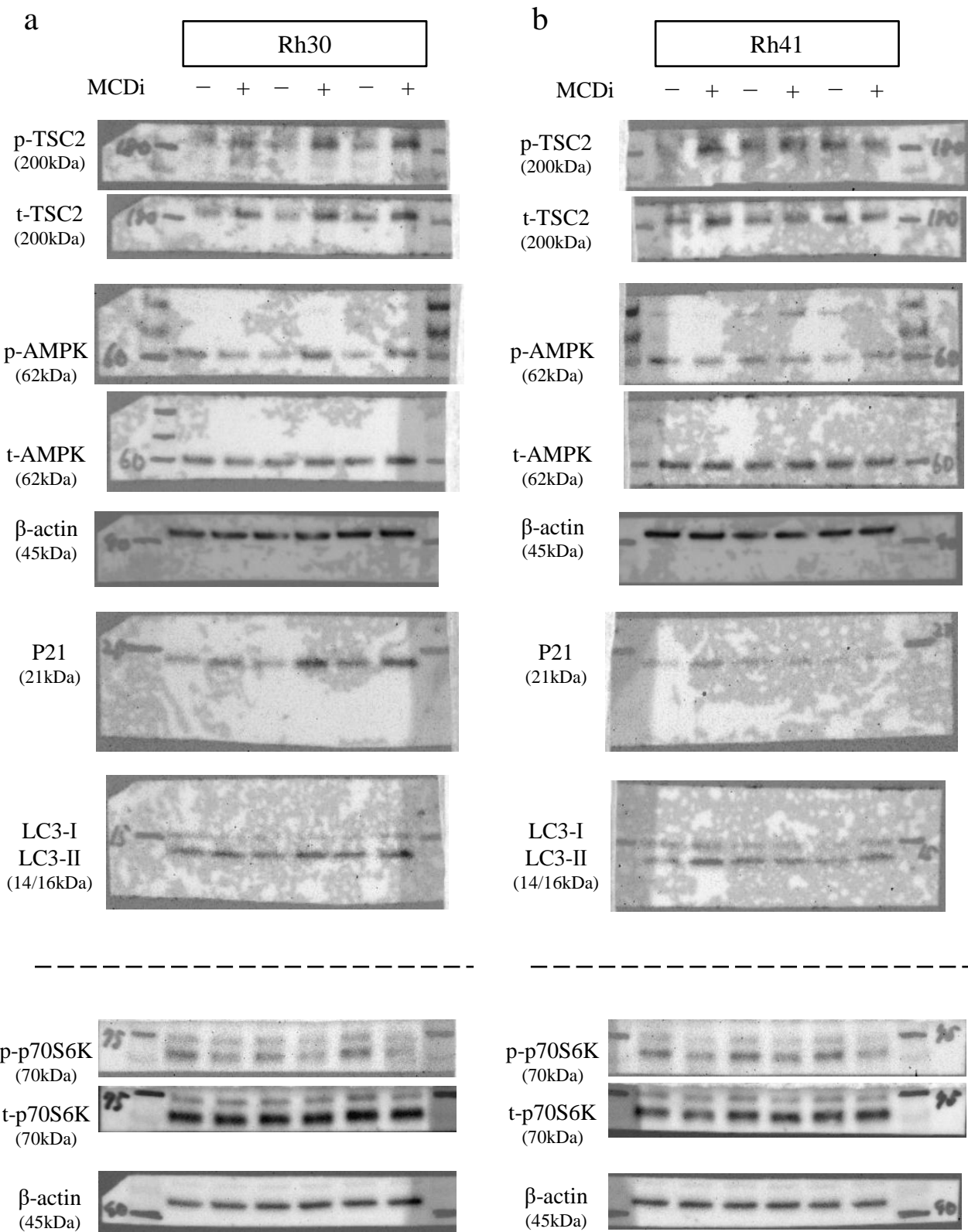

**Figure S1(2).**

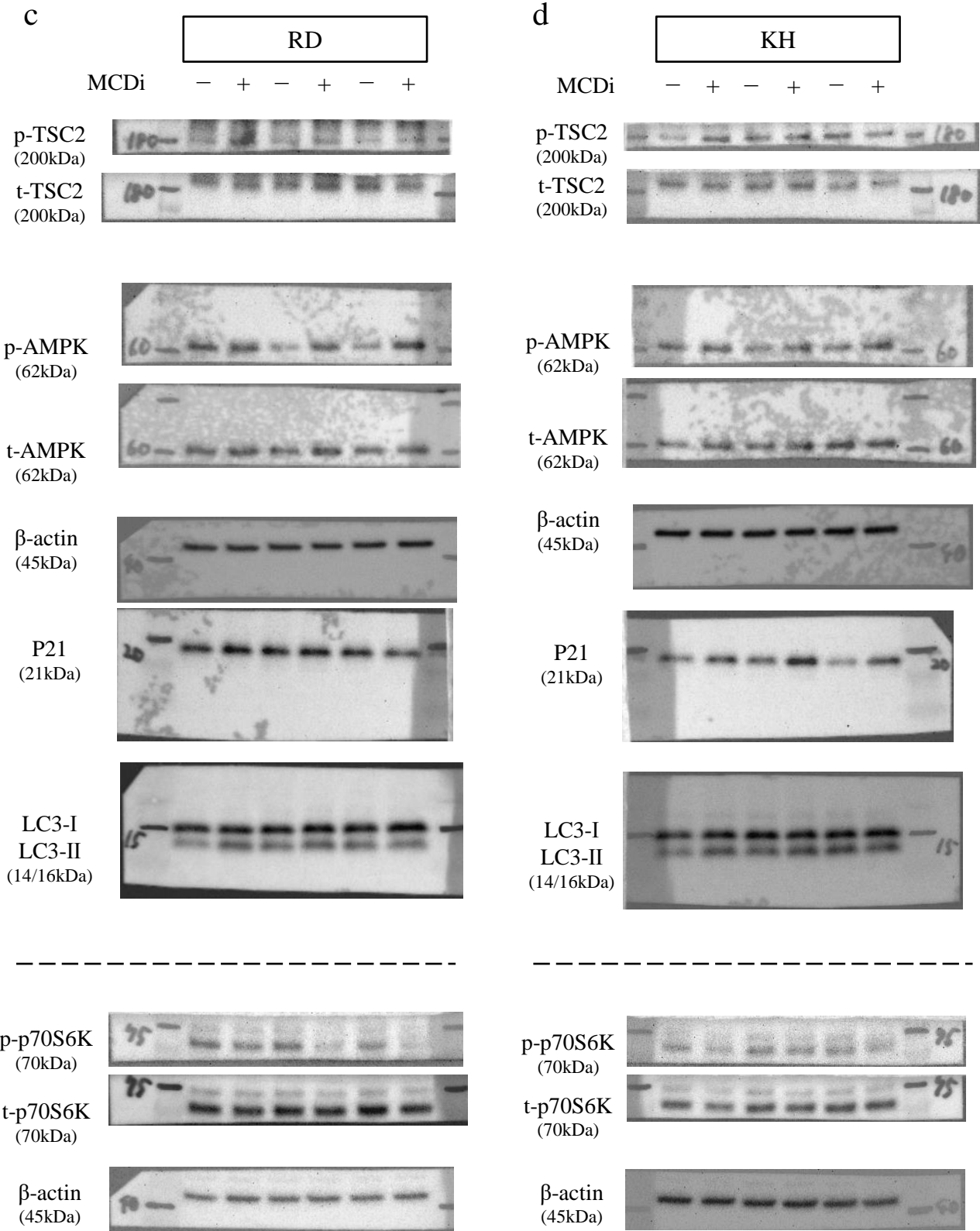

**Figure S2.**

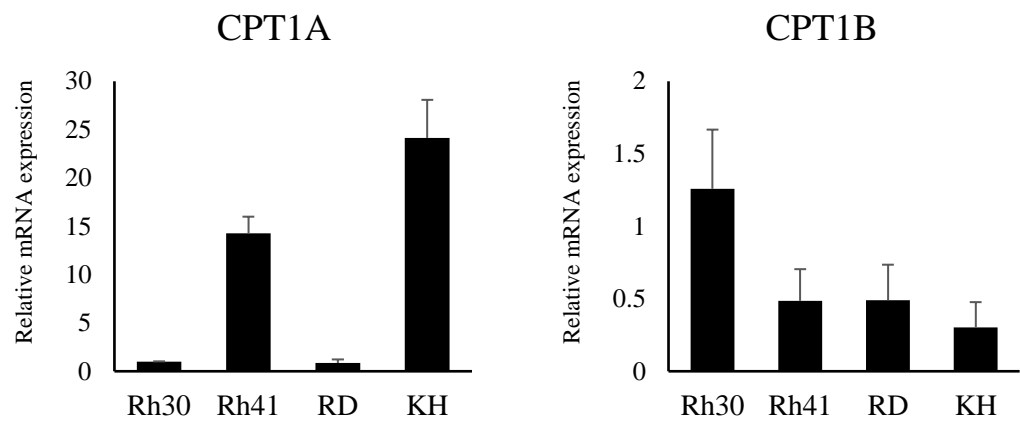

Supplement: Supplementary file 1 — Figure S1. Figure S2. [file CAM4-10-6442-s001.pdf]
