# Supplementary material for: Inhibition of lipid metabolism exerts antitumor effects on rhabdomyosarcoma
Source: Cancer Med. 2021 Sep 2;10(18):6442–55. doi: 10.1002/cam4.4185 (PMC8446407; doi:10.1002/cam4.4185)
Supplement: Supplementary file 2 — Table S1. [file CAM4-10-6442-s002.docx]

**Table S1.** Primers used in qRT-PCR (human).

| Genes | Primer sequences | |
| --- | --- | --- |
| *CPT1A* | Forward | TTGGACCGGTTGCTGATGAC |
|  | Reverse | TTCCAGCCCAGCACATGAAC |
| *CPT1B* | Forward | TTCTTCCGCCAAACCCTGAA |
|  | Reverse | ACAGACTCTAGGTAAGCCCA |
| *G6PD* | Forward | ACGACGAAGCGCAGACAG |
|  | Reverse | AGCCCACGATGAAGGTGTTT |
| *LDH-A* | Forward | ACGTCAGCATAGCTGTTCCA |
|  | Reverse | TGGAACCAAAAGGAATCGGG |
| *PDHK4* | Forward | ACCCAAGCCACATTGGAAGC |
|  | Reverse | AACACTCAAAGGCATCTTGGAC |
| *β actin* | Forward | GATTCCTATGTGGGCGACGA |
|  | Reverse | AGGTCTCAAACATGATCTGGGT |
